# Full Polymer Dielectric Elastomeric Actuators (DEA) Functionalised with Carbon Nanotubes and High-*K* Ceramics

**DOI:** 10.3390/mi7100172

**Published:** 2016-09-23

**Authors:** Tilo Köckritz, René Luther, Georgi Paschew, Irene Jansen, Andreas Richter, Oliver Jost, Andreas Schönecker, Eckhard Beyer

**Affiliations:** 1 Chair of Laser and Surface Technology, Technische Universität Dresden, 01069 Dresden, Germany; 2 Chair of Polymeric Microsystems, Technische Universität Dresden, 01069 Dresden, Germany; reneluther1@gmail.com (R.L.); georgi.paschew@tu-dresden.de (G.P.); 3 Fraunhofer-Institut für Werkstoff- und Strahltechnik (Fraunhofer IWS), Winterbergstraße 28, 01277 Dresden, Germany; irene.jansen@iws.fraunhofer.de (I.J.); oliver.jost@iws.fraunhofer.de (O.J.); eckhard.beyer@iws.fraunhofer.de (E.B.); 4 Fraunhofer-Institut für Keramische Technologien und Systeme (Fraunhofer IKTS), Winterbergstraße 28, 01277 Dresden, Germany; andreas.schoenecker@ikts.fraunhofer.de

**Keywords:** electroactive polymers, sensors, actuators, conductive polymers, technologies for polymeric microsystems, full polymer actuator, electromechanical characterization, polydimethylsiloxane

## Abstract

Dielectric elastomer actuators (DEA) are special devices which have a simple working and construction principle and outstanding actuation properties. The DEAs consist of a combination of different materials for the dielectric and electrode layers. The combination of these layers causes incompatibilities in their interconnections. Dramatic differences in the mechanical properties and bad adhesion of the layers are the principal causes for the reduction of the actuation displacement and strong reduction of lifetime. Common DEAs achieve actuation displacements of 2% and a durability of some million cycles. The following investigations represent a new approach to solving the problems of common systems. The investigated DEA consists of only one basic raw polymer, which was modified according to the required demands of each layer. The basic raw polymer was modified with single-walled carbon nanotubes or high-*k* ceramics, for example, lead magnesium niobate-lead titanate. The development of the full polymer DEA comprised the development of materials and technologies to realise a reproducible layer composition. It was proven that the full polymer actuator worked according to the theoretical rules. The investigated system achieved actuation displacements above 20% regarding thickness, outstanding interconnections at each layer without any failures, and durability above 3 million cycles without any indication of an impending malfunction.

## 1. Introduction

**Figure 1:**
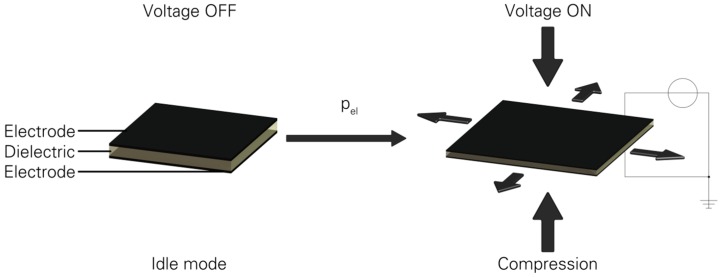
Scheme of the operating principle of a dielectric elastomer actuator (DEA) [3,4,15].

The initiative taken by governments to reduce emissions of CO_2_ during the production process and for the whole lifetime of products increases the interest in new and unconventional solutions. Therefore, industrial companies are finding ways to reduce pollution emissions and save valuable energy. Dielectric elastomer actuators (DEA) represent a promising field of research. The first investigated DEA was published by Röntgen at 1880 and since 1992 DEAs have been back in the focus of researchers and industrial operators as part of further investigations [1,2,3,4]. DEAs are not only applicable as actuators but also as sensors or energy harvesters [5,6,7,8,9,10,11,12,13,14]. DEAs consist at least of three layers, whereby a flexible dielectric is covered on both sides with compliant electrodes [3]. The operating principle of a DEA is based on the electrostatic pressure *p*_el_ = ε_0_·ε_r_·*U^2^*·*z*^−2^, which causes the displacement of the actuator *s_z_* = −ε_0_·ε_r_·*U^2^*·*Y*^−1^·*z*^−2^ [3,4]. The specific values of the past equations are the relative dielectric coefficient ε_r_, thickness of the dielectric *z*, elastic modulus *Y* of the dielectric layer, permittivity of vacuum ε_0_ and driving voltage *U*. Figure 1 visualise the operating principle of DEAs. 

The main limiting factors of conventional DEAs are the achievable displacement and the durability. The layer composition is affected by significant differences regarding mechanical properties like elastic modulus and strain between the electrode and dielectric. Conventionally, electrodes consist of thin metallic layers, graphite grease, carbon black grease or carbon nanotubes (CNTs) [16,17,18,19,20,21]. The average durability for such DEAs with a dielectric layer consisting of polydimethylsiloxane (PDMS) is approximately 10^8^ cycles of actuation [16,20,22]. However, those electrodes consisting of grease containing graphite, silver and carbon achieve a higher durability like thin metallic electrodes [16,20,22]. Additionally, the adherence of the conventional electrodes to the dielectric and the brittleness of the electrodes are further limiting factors. Danfoss PolyPower^®^ (Danfoss Polypower A/S, Nordborg, Denmark), which is the most common DEA material, reduces these problems by structuring the surface of the dielectric [18,23]. DEAs consisting of Danfoss PolyPower^®^ with a back-to-back configuration achieves a maximum displacement of 2% with an applied field strength of 31.25 kV/mm, which corresponds to a driving voltage of 2.5 kV [18,24].

Furthermore, another approach to solve the problems of conventional DEAs is to build the whole actuator with only one basic raw material to achieve a full polymer DEA [25]. The required characteristics for dielectric and electrode layers are achieved by a modification of the basic raw polymer using respective fillers. Full polymer actuators will be able to address the incompatibilities of the DEAs, whereby durability and system performance will increase.

## 2. Experimental Section

### 2.1. Materials

**Figure 2:**
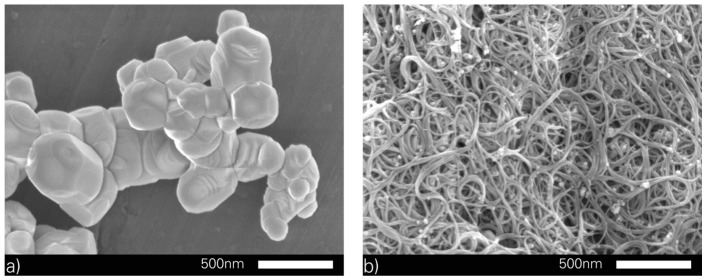
Scanning electron microscopy (SEM) image of (**a**) the perovskite lead magnesium niobate-lead titanate (PMN-PT) produced by Fraunhofer IKTS [15]; and (**b**) single-walled carbon nanotubes (SWCNTs) produced by Fraunhofer IWS [15].

The selection of the basic raw polymer is based on fundamental pre-investigations of two-components and additive curing PDMS. Three of the most important brands of PDMS were compared and analysed as a dielectric for DEAs. Here, the DowCorning Sylgard^®^ 184 (DowCorning Corp., Midland, MI, USA), Momentive RTV615 (Momentive Performance Materials GmbH, Leverkusen, Germany) and Wacker Elastosil^®^ RT 625 (Wacker Chemie AG, München, Germany) were evaluated regarding their processability, reproducibility of the thickness and their mechanical parameters of the polymer layers. Further parameters like viscosity, pot-life and time for cross-linking were characterised. These factors are important for the modification and production of full polymer DEAs. The DowCorning Sylgard^®^ 184 satisfied the requirements at all points of interest and was chosen as basic raw polymer. DowCorning Sylgard^®^ 184 was purchased from Arrow Electronics Inc. (LS Venlo, The Netherlands) and Biesterfeld Spezialchemie GmbH (Hamburg, Germany) as kit [26]. The material used as electrical conductive filler were single-walled carbon nanotubes (SWCNTs) synthesised by Fraunhofer IWS, Dresden. The ferroelectric perovskite lead magnesium niobate-lead titanate (PMN-PT), which was used as high-*k* dielectric filler, was synthesised by Fraunhofer IKTS, Dresden. Figure 2 shows scanning electron microscopy (SEM) images of the used (a) PMN-PT; and (b) SWCNTs. Additionally, common chemicals were applied during the whole investigation process. The preparation and cleaning of surfaces, devices and tools were done with ethanol (denatured), acetone, isopropanol and dichloromethane, which were obtained from Merck KGaA (Darmstadt, Germany).

### 2.2. Synthesis of Single-Walled Carbon Nanotubes

The SWCNTs were synthesised with a pulsed DC arc process. The process is based on a multi-component catalyser consisting of a mixture of cobalt, nickel, iron and molybdenum. The targets were vaporised, transported through the furnace by a gas flow and later on absorbed at a water-cooled collector. The values of the physical vapour deposition (PVD) process were a current of 100 A, a voltage of 50 V, a pulse-width of the pulsed DC arc at the range of dozens of ms, a furnace temperature of 1000 °C and a gas pressure of 100 mbar [27]. 

**Figure 3:**
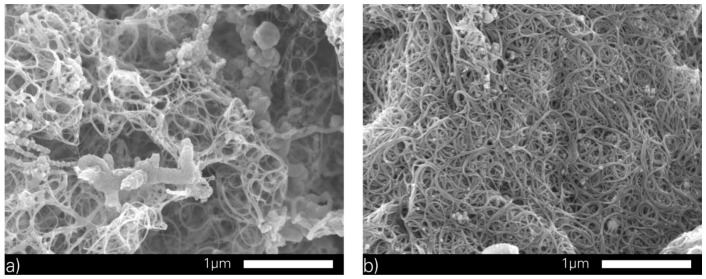
(**a**) SEM image of the produced material containing carbon, particles of the catalysts and SWCNTs; and (**b**) purified material.

The produced soot consists of 20 wt % of particles from the catalysts and 80 wt % of carbon. This carbon contains 40–60 wt % of SWCNTs and these were divided into semiconducting and metallic SWCNTs. The content of the metallic SWCNTs was approximately 65 wt %. Additionally, the soot was purified by a wet-chemical process with HNO_3_ and H_2_O_2_. The purification with HNO_3_ eliminates the metallic particles while the H_2_O_2_ eliminates the graphite. Figure 3 shows (a) the unpurified; and (b) purified SWCNT material. Finally, the purity of SWCNTs powder was approximately 98% of carbon content and only a few catalytic particles like nickel and cobalt. The SWCNTs show an average diameter of 1.25 nm [27,28,29].

### 2.3. Synthesis of Ferroelectric Perovskite PMN-PT

The synthesised perovskite lead magnesium niobate (PMN), which can be assigned to the ferroelectric perovskites, will be stabilised with perovskite lead titanate (PT). Additionally, the PT decreases the curie-temperature to the range of room temperature. Thereby, PMN-PT exhibits the highest achievable permittivity at room temperature with approximately 30.00 and is well suited to high-*k* ceramics. The composition of the used PMN-PT was 0.67 Pb_3_MgNb_2_O_9_-0.33 PbTiO_3_. The resulting powder was only calcinated and not sintered [30,31,32]. 

### 2.4. Design and Fabrication of the Elastomer Actuators

#### 2.4.1. Design of the Elastomer Actuators

The displacement of the actuator was characterised by using DEAs with a three layer composition and circular electrodes. Additionally, DEAs with a three layer composition and rectangular large-scale electrodes were used for investigating the durability. For such DEAs, the number of layers was increased up to 11 layers, in order to demonstrate the performance of full polymer multilayer DEAs as weightlifter and artificial muscle.

#### 2.4.2. Modification of the Basic Raw Material

The modification of the basic raw polymer depends on the type of filler, morphology and kind of agglomeration. Figure 2 visualises the (a) PMN-PT; and (b) SWCNTs, which were used for the modification of the PDMS, and shows the differences regarding their morphology and agglomeration. The PMN-PT is nearly spherical, does not build strong agglomerates and needs only a homogenisation. Therefore, the integration was done by using a dual asymmetric centrifuge, produced by Hauschild Engineering & Co. KG (Hamm, Germany). The SWCNTs are tube-like with a high aspect ratio, build strong agglomerates induced by their reactivity and need a deagglomeration and homogenisation. This was realised by a multistage process. At first, a homogenous premix was built by using the dual asymmetric centrifuge. Subsequently, the deagglomeration and homogenisation was done by a stepwise processing at a three roll mill, produced by EXAKT Advanced Technologies GmbH (Norderstedt, Germany) [33,34,35,36].

#### 2.4.3. Fabrication of Single Dielectric and Electrode Layers

The investigations into the dielectric layer were based on two approaches. The first approach was to investigate the unmodified basic raw polymer, as the dielectric properties of the chosen PDMS are promising. The second approach was to investigate the modification of the basic raw polymer with PMN-PT to increase the actuation properties and to reduce the driving voltage. The filler content of the dispersions for the electrode layer was varied up to the highest amount of 3.0 wt % of SWCNTs to achieve the required electrical conductivity.

**Figure 4:**
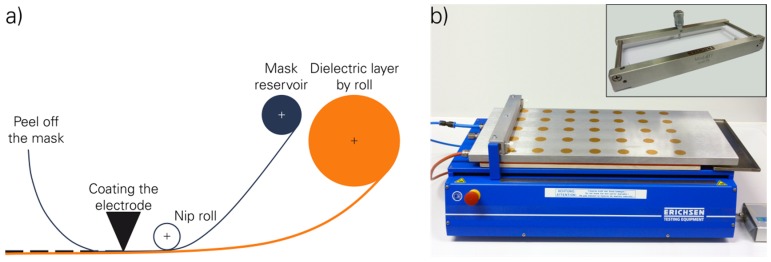
(**a**) Fabrication process for the DEA (method 2) [15]; and (**b**) the COATMASTER 509 MC and the MULTCATOR 411.

The fabrication of the layers began with the addition of the desired curing agent ratio to the different dispersions. This was mixed together by using the dual asymmetric centrifuge for 3 min at 3000 rpm. This material was used to applicate the film onto a carrier substrate. Previous investigations showed that float glass was a promising substrate because it has the lowest surface roughness and offered a sufficient anti-adhesion to the PDMS. The application was done with a COATMASTER 509 MC and a MULTCATOR 411, both produced by Erichsen GmbH & Co. KG (Hemer, Germany), which is shown in Figure 4b. The thickness of each layer was adjustable between 0 and 1000 µm by the coating knife. The width was limited to 150 mm and the length to 400 mm.

#### 2.4.4. Fabrication of the Layer Composition of DEA

Generally, the fabrication process of the different designs for DEAs was investigated for two different methods. The technological key factors of the investigation are the production of DEAs with thin dielectric and electrode layers without inhomogeneities and imperfections, a high variability of geometry and the opportunity to transfer, scale up and automate the technology. The main aim was to produce DEAs with a high reproducibility and durability, which enables long-term stability and outstanding actuation parameters. The fabrication was performed with the previously described methods for the production of single layers, since any variations of the fabrication process can influence the final material characteristics. Method 1 was based on previously produced and cross-linked electrode and dielectric layers, which were interconnected with an additional basic raw polymer adhesive layer. The major disadvantages of method 1 are the additional layer for the interconnection and a poorly heat-supported curing due to the different thermal expansion coefficients of the layers. In method 2, which is the most promising approach, the dielectric layers were previously produced and cross-linked and the electrodes were directly coated on the surface. The final shape of the electrode layer including the elastic and conductive paths was realised by masks. Multi-layer set-ups shall be stacked under wet or at not completely cross-linked conditions. Figure 4a visualises the fabrication process and (b) the used devices.

### 2.5. Analytical Methods

#### 2.5.1. Rheology Properties of Dispersions

The rheological properties of the dispersion were investigated to evaluate the modification, type of fluid and processability of the dispersion modified with the nanoscale fillers. The main values used for the evaluation were the complex viscosity, storage and loss modulus related to the angular frequency. These factors may be used to determine the type of fluid and the rheological percolation threshold. The fluids are subdivided into Newtonian, shear-thickening or pseudoplastic character. As a consequence, the change in the processability is indicated by the rheological behaviour and can be adjusted to ensure a stable process. The rheology behaviour was measured by a digital rheometer C-VOR (Malvern Instruments GmbH, Herrenberg, Germany) [37,38]. 

#### 2.5.2. Electrical Properties of the Elastomer Films

The dielectric properties were investigated by means of the electrical breakthrough for the dielectric with the investigated polymer electrodes and as comparison with sputtered electrodes of gold. The diameter of the electrodes was 2.0 cm. The destructive testing was done with a HIPOT Tester with a maximum DC voltage of 12 kV, which was produced by Sefelec GmbH (Ottersweier, Germany) [39]. 

The electrical values were classified into two divisions with a threshold of 10^5^ Ohm·m, whereby the conductive and semi- and non-conductive materials were detached. The measurement above the threshold was done with an Electrometer/High Resistance Meter 6517B and Resistivity Test Fixture 8009, both produced by Keithley Instruments Inc. (Cleveland, OH, USA) [40,41,42,43]. The characterisation below the threshold was done with a Keithley Digital Multimeter 2000 and self-made fixture based on the four-wire-measurement [44,45,46,47]. The samples for the Resistivity Test Fixture 8009 are quadratic with a dimension of 6.5 cm. The samples used for the four-wire-resistivity measurement are 7 cm long and 3 cm wide. Both were 140 mm thick.

#### 2.5.3. Mechanical Properties of Films and Compounds

The mechanical characterisation of the different layers was done to investigate the adjustment of the mechanical parameters. The test was used to investigate the elastic modulus and the shear tension together, which is based on the DIN EN ISO 527 norm [48,49] and ASTM D 638-14 [50]. The testing speed was 1 mm/min at the elastic modulus and 10 mm/min at the shear tension [48,49,50]. The used probes were 13.0 cm long, 2.5 cm wide and 0.04 cm thick.

The mechanical characterisation for the layer composition was done by peeling tests to examine the interconnection of the layers, which was based on DIN EN ISO 11339 [51] and ASTM D 1876-01 [52]. The testing speed was 10 mm/min, the probes were 13.0 cm long, 2.5 cm wide and 0.04 cm thick and the peeling distance was 6.0 cm [51,52]. Both tests were done with the testing machine Zwick/Roell Z050 (Zwick GmbH & Co. KG, Ulm, Germany) and the shear tension test was supported by the optical measurement system ARAMIS 5M (version 6.3, Gesellschaft für Optische Messtechnik mbH, Braunschweig, Germany) to characterise the real strain.

#### 2.5.4. Actuator Properties

The characterisation of the displacement of the actuators is based on the principle of a mechanical thickness shear crystal, which was done for three layer actuators with circular electrodes [53]. Therefore, a high-precision experimental rig was designed, constructed and realised. The displacement of different actuators was determined by using an interferometric measuring system to evaluate the thickness variation of the DEA regarding driving voltage. In addition, an interferometer ZMI^TM^ 7702 by Zygo Corp. (Middlefield, CT, USA), a power supply HM8142 by Hameg Instruments GmbH (Germany) and a high-voltage amplifier 609B by Trek Inc. (Lockport, NY, USA) was used [54,55,56]. Additional information is shown in Section 1 of Supplementary Material.

The characterisation for large-scale DEAs was done according to the pure shear measurement described on [57] for the constant force method. This set-up was used to investigate the durability and displacement for three layer and multilayer DEAs as an artificial muscle acting as a weightlifter. The actuators were wound around two separate hollow cylinders and a tubeless DEA was built, which was only fixed by squeezing. The displacement was measured by elongation of the weight with the laser interferometer. Additional information is shown in Section 2 of Supplementary Material.

The investigations into lifetime were done for electro-mechanical stress induced by a determined driving voltage and a frequency. Additionally, the lifetime of the DEAs was investigated for mechanical stress induced by a determined elongation and a frequency, whereby a cyclic measurement of the electrical strength was executed.

## 3. Results and Discussion

### 3.1. Fundamental Characterisation of the Basic Raw Polymer

The mechanical properties like stress–strain behaviour and the elastic modulus—just as the dielectric strength of the unmodified basic raw polymers is a very important factor—can be controlled by changing the proportions of the curing agent ratio and the curing conditions. These factors are useable for the mechanical adjustment to achieve a correlation of the dielectric and electrode layers. On the other hand, it is possible to achieve reproducible parameters. These material properties of the silicone were investigated for a curing agent ratio of 8:1 up to 21:1. The recommended curing agent ratio for the PDMS was 10:1 [26]. The elastic modulus and stress-strain-behaviour can be regulated by a surplus or deficit of hardener. Hereby, a range of the elastic modulus between 2.72 MPa (8:1) and 0.52 MPa (21:1) was adjustable. The achievable strain was also adjustable between 80% and 170%. The variation of the curing agent ratio does not influence the relative permittivity and an average value of 3.44 was investigated. The dielectric strength appeared to have an influence through its variation and the lowest dielectric strength was 78.8 kV/mm. Figure S2 shows the investigated stress–strain behaviour, relative permittivity and dielectric strength corresponding to the curing agent ratio.

### 3.2. Rheology of the Dispersions

The rheological properties of the basic raw polymer were affected by the modification with the nanoscale fillers. The change of the complex viscosity may become a crucial value for the material development regarding to the processability of the dispersions to monolithic full polymer DEAs. The morphology of the particles showed different behaviours regarding the affectation of the rheological behaviour, shown in Figure S3. The addition of 66.6 wt % of PMN-PT caused an increase in the complex viscosity from 4.5 Pas up to 9.4 Pas, whereby 3.0 wt % of SWCNTs reached 43.3 kPas, which can be explained by the aspect ratio. PMN-PT do not build strong interconnected networks in contrast to the SWCNTs. The dispersions containing PMN-PT showed a Newtonian behaviour and dispersions containing SWCNTs a shear thinning behaviour: in this case, a reduction of the complex viscosity may cause by an increased shear exposure. The rheological percolation threshold and change of the microstructure can be characterised by the storage or loss modulus for anisometric fillers, described by [38]. The investigations showed a percolation threshold of 0.5 wt % and a change of the microstructure. Figure S4 shows the investigated storage and loss modulus vs. the angular frequency. Additional information is presented in [33,34,35,36].

### 3.3. Mechanical and Electrical Properties of the DEA

#### 3.3.1. Electrode Layers

The aim was to achieve the necessary electrical conductivity of the basic raw polymer by the integration of SWCNTs. The requirements to the electrical conductivity depend on the operating field of the further actuator. A high-dynamic operating of DEAs requires a high conductivity and under quasistatic conditions lower conductivities are required. The investigations of [58,59] showed that a sheet resistance significantly higher than 10 kOhmsq is insufficient for the electrode layers for DEAs. 

**Figure 5:**
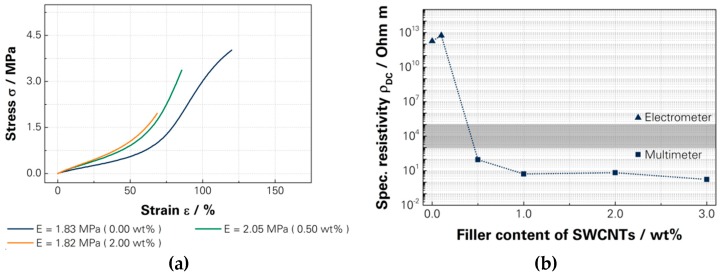
(**a**) Shift of the stress–strain behaviour [15]; and (**b**) the specific conductivity through the modification of SWNCTs.

The modification of the basic raw polymer with SWCNTs had an influence on the mechanical values, which was negligible. The elastic modulus fluctuates in the range between 1.82 MPa and 2.05 MPa. The stress was reduced from 4.0 MPa to 1.96 MPa and the strain from 120% to 70% by the addition of 2.0 wt % of SWCNTs. Additionally, a counteracting effect should be possible through the adjustment of the basic raw polymer. Figure 5a shows the influence of the mechanical values.

The reduction of the specific resistivity achieved at the percolations threshold of 0.5 wt % of SWCNTs is nearly 90 Ohm·m and for 3.0 wt % 1.7 Ohm·m, shown in Figure 5b. The basic raw polymer has a specific resistivity above 10^14^ Ohm·m. According to [58,59], the threshold was reached between 2 wt % and 3 wt %, whereby 3.0 wt % scored 5.4 kOhm. Therefore, the filler content was determined to be 3.0 wt %. 

#### 3.3.2. Dielectric Layers

The modification of the basic raw polymer with PMN-PT was focused on increasing the relative permittivity without having a strong influence on the other parameters like the elastic modulus, stress–strain behaviour or the complex viscosity. The first detected threshold was the processability of dispersions with a filler content beyond 50 wt %. The particles accumulated in the coating knife gap and prevented the production of homogeneous and reproducible dielectric layers. Furthermore, the elastic modulus was significantly changed from 1.83 MPa for unfilled PDMS to 3.06 MPa for a filler content of 50 wt % of PMN-PT. The stress showed a slight reduction by the addition of 33.3 wt % of PMN-PT but again an increase to 3.8 MPa for 50 wt % of PMN-PT. The strain was reduced from 120% to 80%. If necessary, further adjustments of the mechanical parameters are possible. The relative permittivity was increased from 3.2 to 4.6 by the addition of 33.3 wt % and to 6.8 with 50.0 wt %. The dielectric strength was for each step of modification above 80 kV/mm. The plots are shown in Figure S5.

#### 3.3.3. Comparison of Full Polymer and Metallic Electrodes for DEAs

The production process of the DEAs with sputtered electrodes is completely different to the fabrication of the full polymer DEAs. Therefore, the influence of the dielectric parameters was investigated for unmodified dielectrics and for dielectrics modified with PMN-PT. The dielectric benefits stemming from the polymer electrodes and a higher dielectric strength can be observed, which was leastwise 30 kV/mm higher compared to the values achieved with gold electrodes. The process for the deposition of the gold electrode seems to influence the properties of the dielectric layer and the vaporised gold may penetrate the surface, whereby the distance between the electrodes is reduced. Oppositely, the relative permittivity is not shown to have such a significant influence on the values. The investigated difference can be led back to variations of the thickness of the dielectric. Additionally, the influence of different curing agent ratios was also investigated and showed that the polymer electrodes achieve a higher dielectric strength than the sputtered electrodes by at least 40 kV/mm. The variation from 10:1 to 16:1 caused an increase of the dielectric strength from 46.8 kV/mm to 51.14 kV/mm using sputtered electrodes and with polymer electrodes from 84.6 kV/mm to 107.4 kV/mm. In comparison, the full polymer DEAs achieved distinctly higher dielectric strengths than metallic electrodes and Danfoss PolyPower^®^. The plots are shown in Figures S6–S8.

#### 3.3.4. Three-Layer DEA

**Figure 6:**
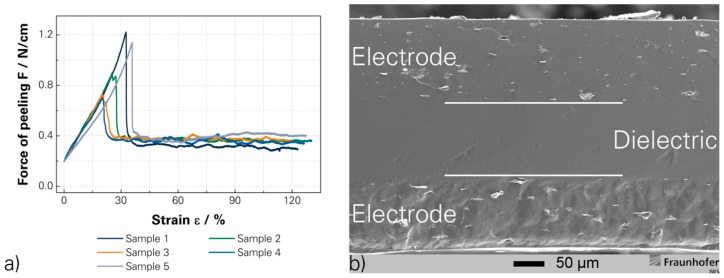
(**a**) Mechanical testing of the peeling force [15]; and (**b**) a cryogenic-fractured SEM image of a three layer DEA [15].

The investigated production technology for the full polymer DEAs was construed as variable and scalable process. The investigated production technology satisfied a high variability of electrode geometries up to thin elastic conductive paths for the electrical connection of the DEAs, which is only restricted by the mask and structuring technology. The produced DEAs exhibit a high reproducibility with variations below 5% regarding to the layer thickness and mechanical properties. Additionally, such DEAs achieved an outstanding stability of the different interconnected layers, which was proven by peel tests and SEM images. The peeling is the worst kind of exposure to the interconnection and peeling forces of 0.4 N/cm were achieved. Additionally, the SEM image of cryogenic-fractured DEAs does not show any trapped air, kissing bonds or some other failures of the interconnection. Therefore, an outstanding durability of the DEAs was achieved and malfunctions caused by the production process and layer interconnection are unexpected. Figure 6a shows the peeling test and (b) a SEM image of a cryogenic-fractured three layer DEA. Figure 6b visualises also a slight re-agglomeration of the SWCNTs along the fracture. The reactivity of the SWCNTs caused the re-agglomeration, which is positive for the electrical conductivity [60,61]. These sub-agglomerates build interconnections between the SWCNTs and reduce the percolation threshold [60,61]. Figure S9 shows three kinds of produced DEAs.

### 3.4. Actuator Properties

#### 3.4.1. Influence of Material and Design Properties

The actuator properties were investigated mainly with DEAs produced by unmodified silicone as the dielectric layer and by 3 wt % SWCNTs modified basic raw polymer as the electrode material. The displacement *s_z_* of the DEAs can be influenced mainly by the relative permittivity, the elastic modulus and the thickness of the dielectric layer. The relative permittivity of the dielectric was adjustable by the addition of PMN-PT, the elastic modulus by the variation of the curing agent ratio or modification with PMN-PT, and the thickness of the dielectric layer can be reduced by the adjustment of the coating knife or further adaptions of the investigated production technology.

#### 3.4.2. Operation Parameters

The investigation of the actuation displacement was done with predetermined operation parameters for driving voltage and times for charging, maximum load, discharging and without load. The displacement was investigated by a short term exposure with several repetitions. The specified times were exactly the same for each driving voltage and only the maximum load was changed. The time for charging and discharging was determined to be 10 s and the time under maximum load was 1 s. Additionally, the maximum displacement for static loadings can be investigated. Here, the time under load was extended to 600 s, which can be observed as static load. The determined regimes are presented in Figures S10 and S11.

#### 3.4.3. Actuation Behaviour

The actuation behaviour was investigated for different configurations of three layer actuators to determine the influence of the curing agent ratio and the modification with PMN-PT of the dielectric layer, different kinds of operation parameters and the durability of the DEAs. The standard configuration of the actuators has an unfilled dielectric with a curing agent ratio of 10:1 and a geometry of (15 × 15 × 0.011) cm, just as electrodes containing 3.0 wt % of SWCNTs with a diameter of 2 cm. Table S1 gives a detailed overview of the actuators used and their configurations. 

**Figure 7:**
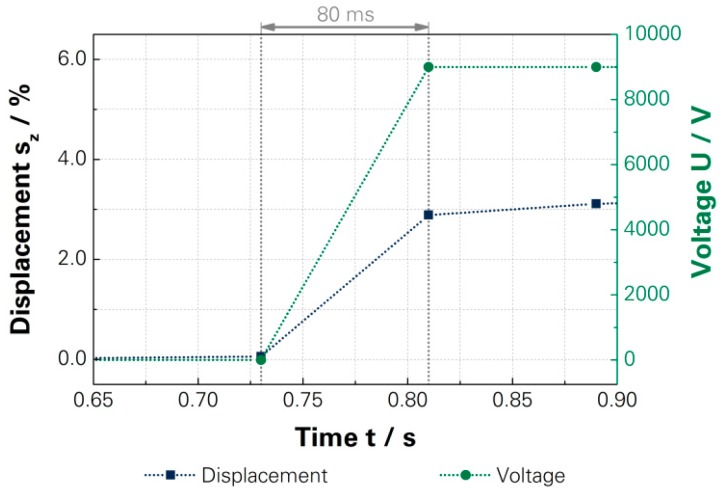
Investigated reaction time of a standard DEA [15]—Table S1: actuator 1.

At first, the reaction time for the standard actuator was investigated for a driving voltage of 9000 V and reached 80 ms, shown at Figure 7. The investigation was limited by the equipment and a further reduction should be possible. Additionally, Figure 7 shows that the main part of the displacement was reached directly after turning on even though a further displacement takes place during the subsequent period. This can be explained through the Mullins effect, whereby the elasticity of the polymer depends on the duration and count of cycles of the acting exposure [62,63,64]. Therefore, the actuation displacement achieved for the short time exposure can differ from a static exposure. The evaluation of short term exposure is done based on the measured displacements regarding the driving voltage for several cycles, which is used for calculating the average displacement. The average displacement of the driving voltages was used to calculate the characteristic curve of the actuator, as presented in Figure S12.

**Figure 8:**
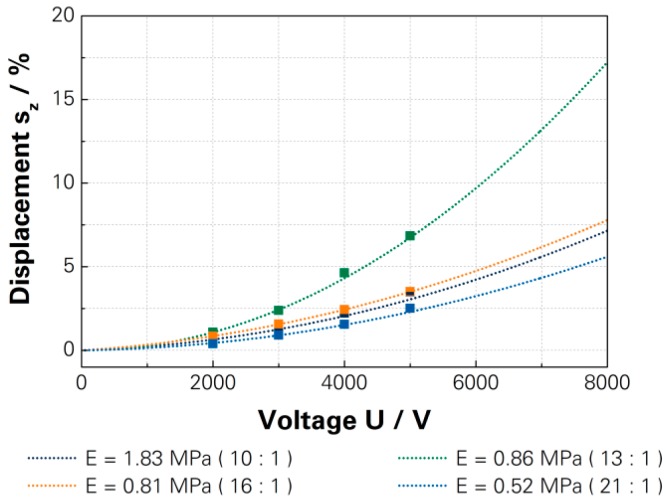
Measured actuation displacements and characteristic curves of full polymer DEA with different elastic modulus [15]—Table S1: actuators 2–5.

The investigation into the influence of the elastic modulus on the actuation displacement involved the examination of four values, adjusted by the variation of the curing agent ratio. The used DEAs had an elastic modulus of 1.83 MPa (10:1), 0.86 MPa (13:1), 0.81 MPa (16:1) and 0.51 MPa (21:1). The dependency of the actuation displacement was proven but the restoring forces significantly limited the increase of the displacement. The reduction of the elastic modulus to 0.81 MPa caused an increase of the displacement but a further reduction of the elastic modulus caused a decrease of the displacement. The DEA with 0.81 MPa still achieved a displacement beyond the standard actuator and the DEA with 0.51 MPa reached the lowest displacements. The DEAs achieved, with a driving voltage of 7000 V, a displacement of 5.7% (1.83 MPa), 13.3% (0.86 MPa), 5.5% (0.81 MPa) and 4.3% (0.51 MPa). Figure 8 shows the measured actuation displacements and calculated characteristic curves for comparison, and the associated investigations are detailed in Figures S13–S16. 

Additionally, the maximum displacement for the DEAs with an elastic modulus of 1.83 MPa (10:1) and 0.86 MPa (13:1) was investigated to represent the difference between the short term actuation and the maximum displacement. Likewise, the maximum displacements showed that the elastic modulus had a strong influence on the displacement. The DEAs achieved, with a driving voltage of 7000 V, a maximum displacement of 5.3% (1.83 MPa) and 19.1% (0.86 MPa). The stiffer DEA does not show a significant difference between the short term actuation and the maximum displacement, while the elastic DEA showed a dramatic difference of 5.9%. This can be explained by the Mullins effect, whereby the softer DEA allowed a greater elastic flow of the material depending on the duration of the acting exposure. Figure S17 shows the maximum displacement regarding the driving voltage for the stiff DEA (a); and the soft DEA (b).

**Figure 9:**
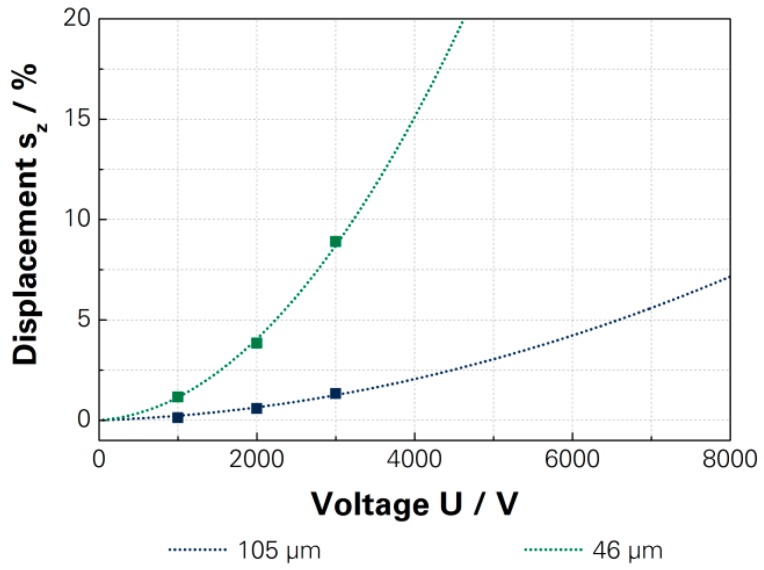
Measured actuation displacements and characteristic curves of full polymer DEA with different thick dielectric layers [15]—Table S1: actuators 2 and 7.

The investigation into the influence of thickness on the actuation displacement of the dielectric layer was investigated for 46 µm and 105 µm. The achieved actuation displacement showed a significant difference between the two DEAs, as shown in Figure 9. The reduction of the thickness can influence the electrical breakthrough, which is why the driving voltage has to be reduced to 3000 V. The determined driving voltage corresponds to an electric field of 71 kV/mm, which is still below the investigated dielectric strength for polymer electrodes of 84.6 kV/mm, and prevents the destruction of the actuator. Therefore, the actuation displacement of the DEA with 105 µm achieved 1.3% and the DEA with 46 µm achieved 8.9%. The actuation displacement for a driving voltage of 5000 V achieved 3.0% for the DEA with 105 µm thickness and 23.1% for the thinner DEA, based on extrapolation of the calculated characteristic curves, which are shown in Figures S18 and S19.

**Figure 10:**
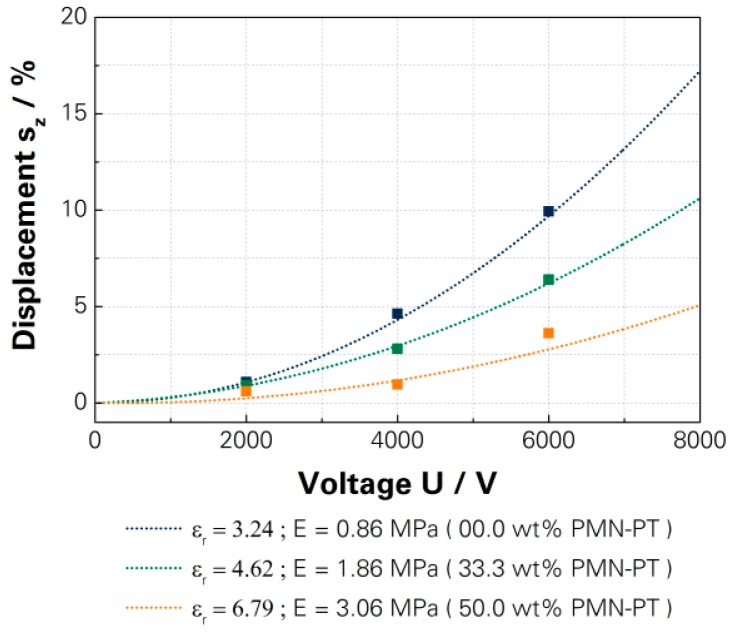
Measured actuation displacements and characteristic curves of full polymer DEA with different relative permittivities [15]—Table S1: actuators 2, 8 and 9.

The influence of the relative permittivity on the actuation displacement was investigated for the PMN-PT modified basic raw polymer. The modification of the PDMS with the PMN-PT increased the relative permittivity but also the elastic modulus. The investigated DEAs achieved a relative permittivity of 3.24 (unmodified), 4.62 (33.3 wt %) and 6.79 (50.0 wt %). The main problem was that the elastic modulus increased faster than the relative permittivity by modification. Therefore, the actuation displacement was reduced. An additional counteracting effect from softening the dielectric layer was not investigated because the presented results based on softened DEAs with a curing agent ratio of 13:1. The achieved displacement with a driving voltage of 7000 V was 13.2% (3.24), 8.2% (4.62) and 3.8% (6.79), which is shown in Figure 10 and is based on the calculated characteristic curves, shown in Figures S20–S22.

**Figure 11:**
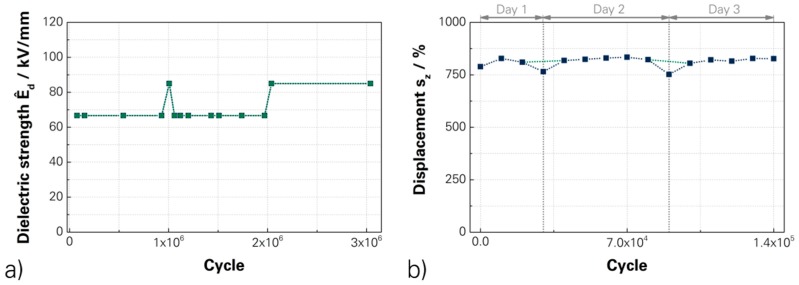
Investigated durability of the fully polymer DEAs for a (**a**) mechanical stress and cyclic measurement [15]; and (**b**) electro-mechanical stress under actuation conditions [15]—Table S1: actuators 10 and 11.

An important factor in the development process was the durability of the full polymer DEAs. The long-term-stability was proven in two ways. Firstly, the DEAs were stressed with 9% elongation and a repetition rate of 1 Hz by mechanical strain. The dielectrical strength was cyclically tested at a threshold of 66.6 kV/mm. At one million cycles and beyond two million cycles, the tested dielectric strength was increased to 85 kV/mm to enhance the demands and the stress. The tests were proven successful for more than three million cycles. Secondly, the DEAs were stressed by electromechanical exposure as a weightlifter at a fixed driving voltage of 5000 V, which corresponded to an electric field of 47.6 kV/mm, and a repetition rate of 4 Hz. The test was successfully driven for 140,000 cycles. The DEAs of both tests did not show any degradations or influences of the material and therefore further cycles will be achievable. Figure 11 showed the investigations for the (a) mechanical; and (b) electromechanical stressing. Additionally, a drift of the actuation displacement was investigated, which was also represented at the electromechanical stress, recognisable for the reduction in displacement every day. Associated investigations show a drift of the displacement, which was measurable up to 2700 cycles but the value of actuation displacement showed only a loss of 10%. Beyond 2700 cycles, there was a displacement of 7.8% with a deviation of 0.07%. Figure S23 shows the curves.

## 4. Conclusions and Outlook

The investigations presented herein show that the newest DEAs have promising actuation properties, which follow the theoretical laws of DEAs. Additionally, full polymer actuators have been proven to have outstanding durability and interconnection between the assembled layers, which is a direct consequence of their monolithic structure. The entire development process for the associated material and technology was successfully completed and the processability and reproducibility were proven. The adjustment of the materials and layers allowed a reproduction of DEAs suitable for further application. The full polymer actuator achieved actuation displacements above 20% regarding thickness and exceeded more than 3 million cycles of mechanical stress without any malfunction or degradation of the DEA. The layers are not fragile and cracked after stress because the used modified silicone is able to resist elongations up to 75% and more.

## Supplementary Materials

The used supplements following are available online at www.mdpi.com/2072-666X/7/10/172. Video S1: The characterization for large-scale DEAs.
