# Supplementary material for: Full Polymer Dielectric Elastomeric Actuators (DEA) Functionalised with Carbon Nanotubes and High-K Ceramics
Source: Micromachines (Basel). 2016 Sep 23;7(10):172. doi: 10.3390/mi7100172 (PMC6189977; doi:10.3390/mi7100172)
Supplement: Supplementary file 2 [file micromachines-07-00172-s002.pdf]

Supplementary Materials: Full Polymer Dielectric Elastomeric Actuators (DEA) Functionalised with Carbon Nanotubes and High-*K* Ceramics

Tilo Köckritz, René Luther, Georgi Paschew, Irene Jansen, Andreas Richter, Oliver Jost, Andreas Schönecker and Eckhard Beyer

1. Actuator Properties

The actuators were clamped on a carrier foil and were fixed by strong magnets. These actuators were added to the experimental rig and arranged by a vertically adjustable pusher, which is guided by a parallel spring. The pusher included a reflecting prism at the top and a lens at the bottom. The lens was used to clamp the dielectric elastomeric actuators (DEA) to the disc underneath and was connected with the prism, which was used to measure the displacement of the actuator. The displacement measuring laser interferometer ZMI™ 7702 from Zygo Corp. (Middlefield, CT, USA) was used to evaluate the variation in thickness of the DEA regarding the driving voltage. Supplement Figure S1a shows a scheme of the measurement set-up and Supplement Figure S1b the high-precision experimental rig.

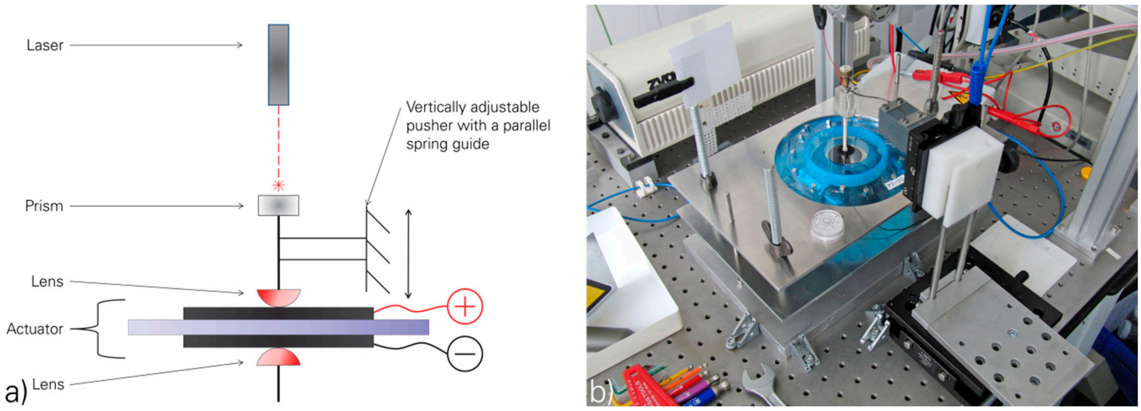

Figure S1. (**a**) Scheme of the developed measurement configuration for evaluating the actuation properties; and (**b**) the real experimental rig [1].

The realised displacement measurement combines a lot of advantages. The actuator was fixed during the measurement by clamping locally between the lenses. The lenses were designed to minimise the contact ratio to the DEA to realise the measurement of only one single spot of the DEA. Additionally, the influence of the surface roughness was reduced. The influence of the effected force and the sinking during the measurement of the pusher was reduced to a minimum by guiding with a parallel spring. Furthermore, a sideways dumping of the prism was avoided by anchoring the parallel spring into a Poly(methyl methacrylate) (PMMA) carrier. The responsivity of the measurement rig was located at the sub-µm range and realised measurements of large displacements without error. The drawbacks of the measurement rig were induced by the variability. The numerous setting options prevent the automation of the measuring process and each measurement was done manually. However, the measurement rig achieved equilibrium results and a high reproducibility.

2. Actuator Properties of Large Scale DEAs

The set-up for the large-scale DEAs was configured for a dimension of the actuator with 200 mm × 100 mm for the dielectric layer and 155 mm × 60 mm for each electrode layer. The set-up may also be used for DEAs with a minimum of three layers but the main aim was the testing of a multilayer actuator. The actuators were wound around two separate hollow cylinders with a

diameter of 50 mm and built therefore a tubeless DEA which was only fixed to the upper and lower tube by squeezing. The set-up was stressed with a weight of 10 kg connected to the lower tube, which was strain-relieved. The displacement of the actuator causes the movement of the weight and this was measured with the laser interferometer ZMI™ 7702 from Zygo Corp.

3. Fundamental Characterisation of the Basic Polymer

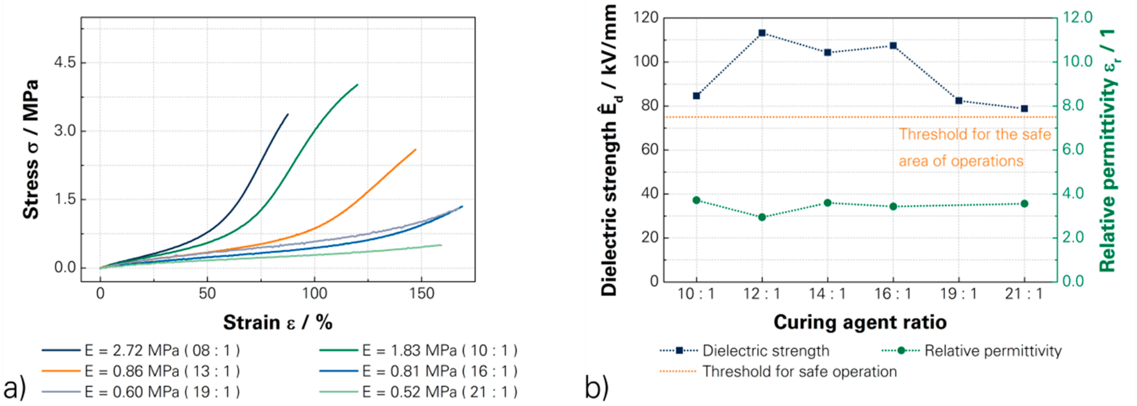

Figure S2. (**a**) Mechanical variability of the basic polymer; and (**b**) corresponding dielectric strength and relative permittivity through the variation of the curing agent ratio [1].

4. Rheology of the Dispersions

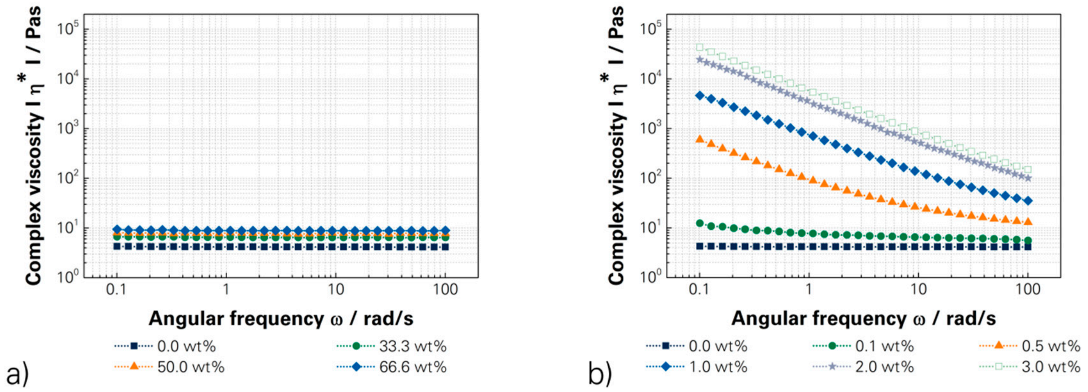

Figure S3. Influence of the modification process with (**a**) perovskite lead magnesium niobate-lead titanate (PMN-PT); and (**b**) single-walled carbon nanotubes (SWCNTs) to the complex viscosity of the basic polymer [1].

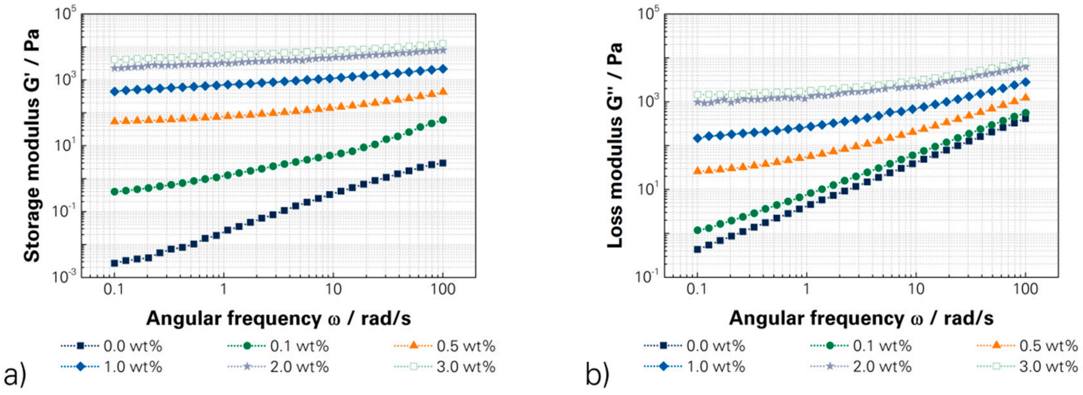

Figure S4. (**a**) Storage modulus; and (**b**) loss modulus of dispersions with SWCNTs [1].

5. Mechanical and Electrical Properties of Dielectric Layers

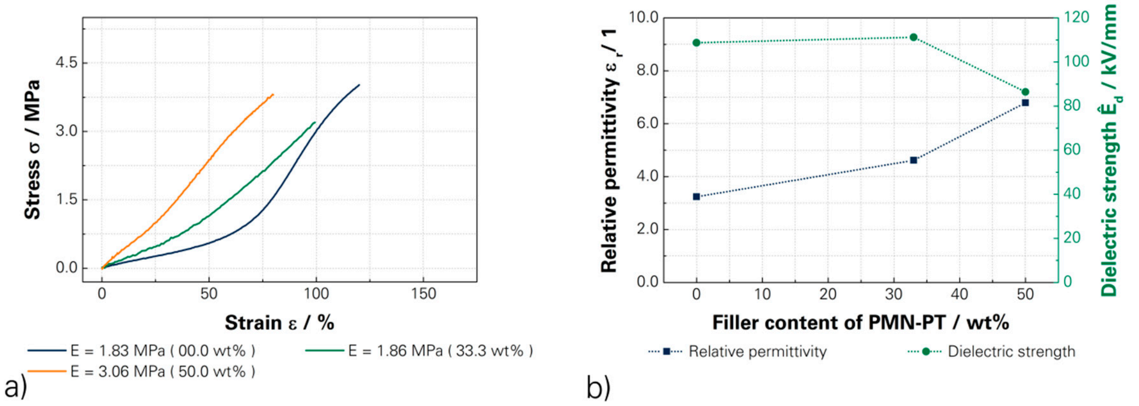

Figure S5. (**a**) Shift of the stress–strain behaviour [1]; and (**b**) the relative permittivity through the modification with PMN-PT.

6. Comparison of Full Polymer and Metallic Electrodes for DEAs

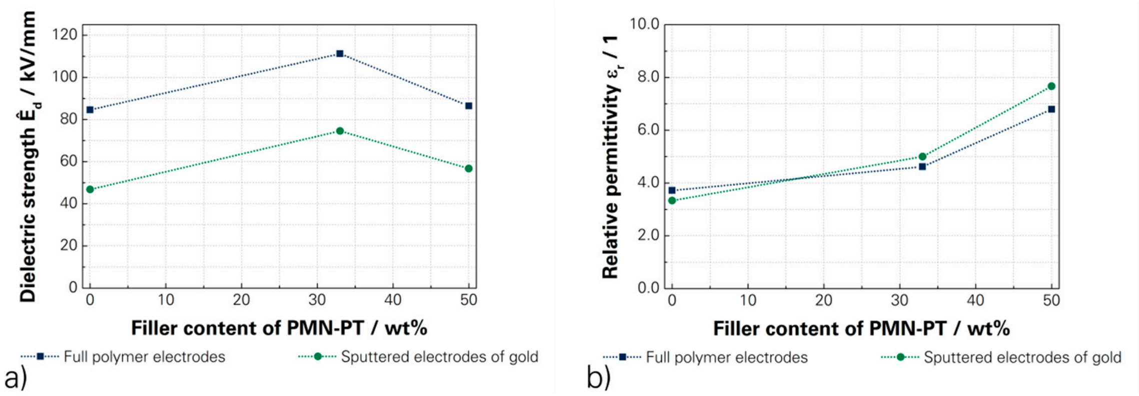

Figure S6. Comparison of conventional sputtered electrodes of gold and full polymer electrodes regarding their (**a**) dielectric strength; and (**b**) relative permittivity for a PMN-PT modified dielectric [1].

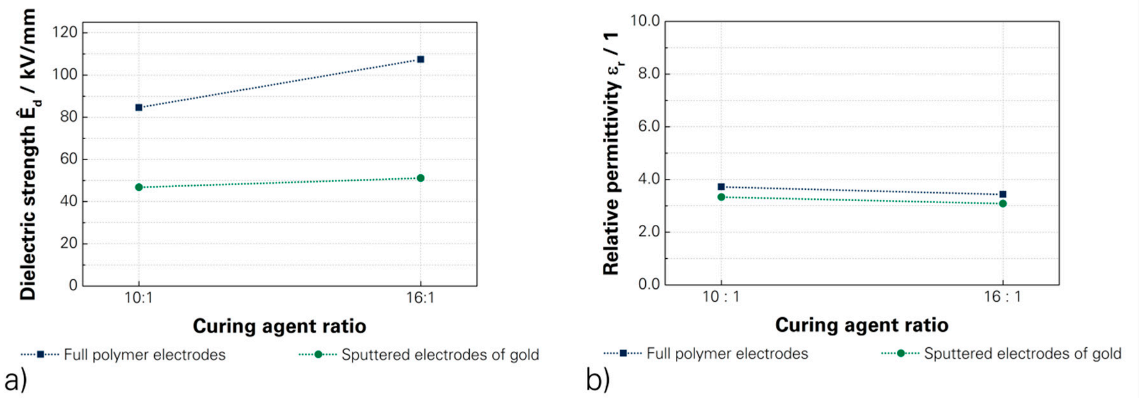

Figure S7. Comparison of conventional sputtered electrodes of gold and full polymer electrodes regarding their (**a**) dielectric strength; and (**b**) relative permittivity for different curing agent ratios of an unmodified polydimethylsiloxane (PDMS) [1].

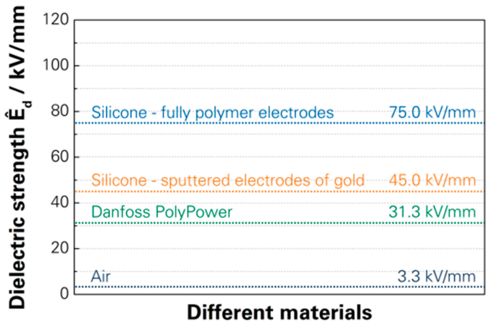

Figure S8. Overview of some typical dielectric strengths [1].

7. Three-Layer DEA

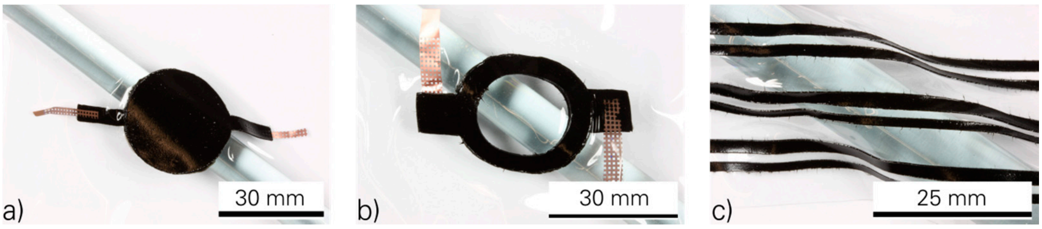

Figure S9. (**a**) Circular DEA with a diameter of 20 mm; (**b**) an annulus DEA with an external diameter of 30 mm and an inside diameter of 20 mm; and (**c**) a set-up of elastic conductive paths with a width of 200 μm, a line spacing of 100 μm and a length of 155 mm.

8. Operation Parameters

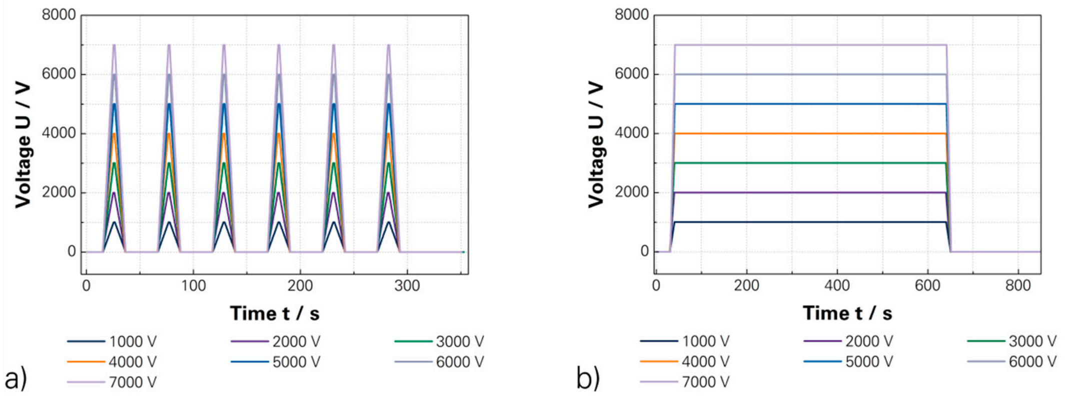

Figure S10. Determined regime for the driving voltage for the investigation of (**a**) the actuation displacement; and (**b**) the maximum displacement between 1000 V and 7000 V [1].

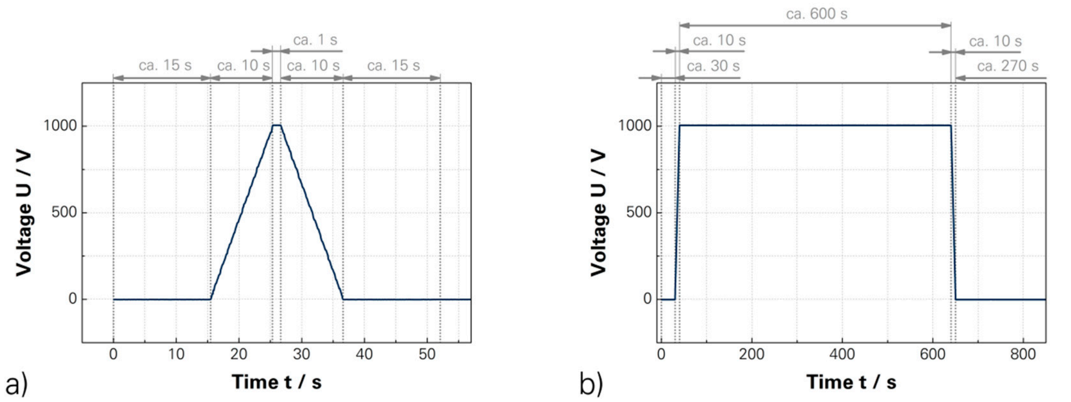

Figure S11. Detailed sequence including the times for the investigation of (**a**) the actuation displacement; (**b**) and the maximum displacement for the driving voltage of 1000 V [1].

9. Actuation Behaviour

Table S1. Overview of the characteristics of the below discussed three layer DEAs.

Characteristic Values of the DEAs	Dielectric Layer											Electrode Layer			
**Actuator**	**1**	**2**	**3**	**4**	**5**	**6**	**7**	**8**	**9**	**10**	**11**	**others**	**3**	**10**	**11**
Filler content/wt %	0.0	0.0	0.0	0.0	0.0	0.0	0.0	33.3	50.0	0.0	0.0	3.0	3.0	3.0	3.0
Curing agent ratio	10:1	10:1	13:1	16:1	21:1	10:1	10:1	10:1	10:1	10:1	10:1	10:1	10:1	10:1	10:1
Electrical properties	3.7	3.7	3.3	3.4	3.6	3.7	3.7	4.6	6.8	3.7	3.7	1.7 Ω·m	1.7 Ω·m	1.7 Ω·m	1.7 Ω·m
Elastic modulus/MPa	1.8	1.8	0.9	0.8	0.5	1.8	1.8	1.9	3.1	1.8	1.8	1.8	1.8	1.8	1.8
Thickness/µm	113	105	111	111	97	89	46	88	91	105	105	100	100	50	55
Dimension/cm	15 × 15	15 × 15	15 × 15	15 × 15	15 × 15	15 × 15	15 × 15	15 × 15	15 × 15	18 × 8	20 × 10	∅ 2	∅ 3	∅ 2	15.5 × 6

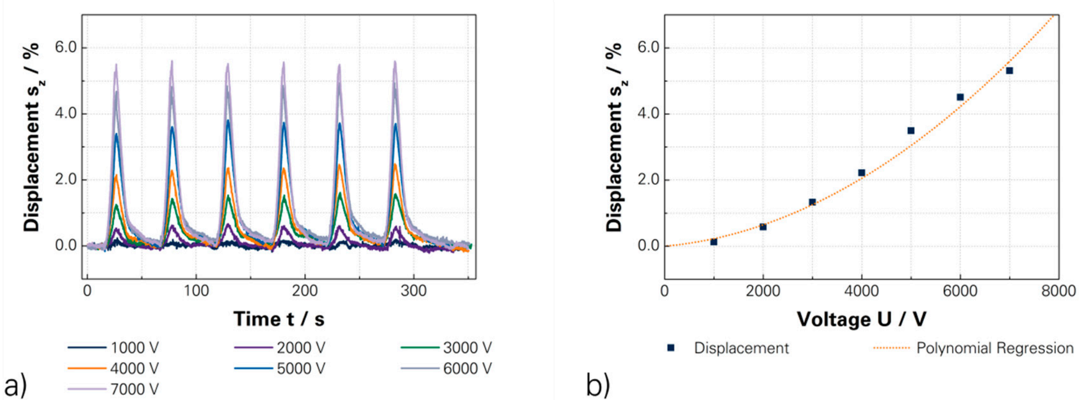

Figure S12. (**a**) Correlation of driving voltage and actuation displacement of a standard DEA; and (**b**) the calculated characteristic curve—Table S1: actuator 1 [1].

10. Actuation Behaviour Regarding Elastic Modulus

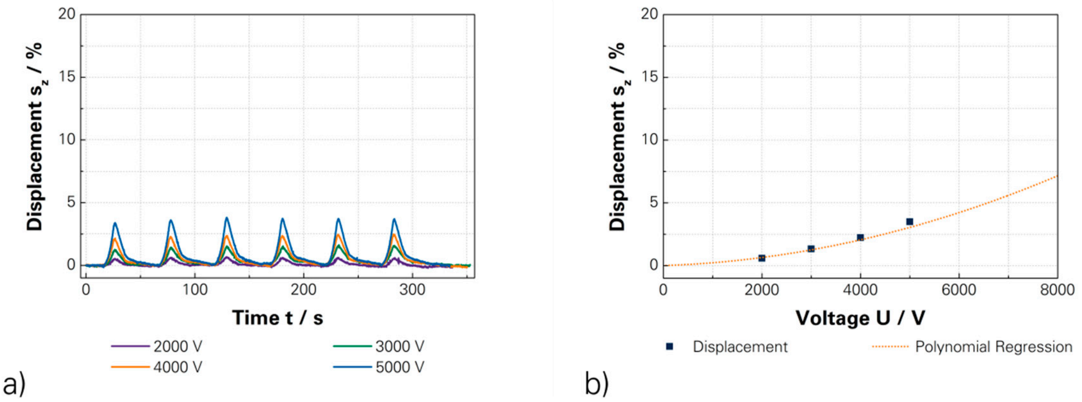

Figure S13. (**a**) Correlation of driving voltage and actuation displacement of a standard DEA (10:1) with an elastic modulus of 1.83 MPa; and (**b**) the calculated characteristic curve—Table S1: actuator 2 [1].

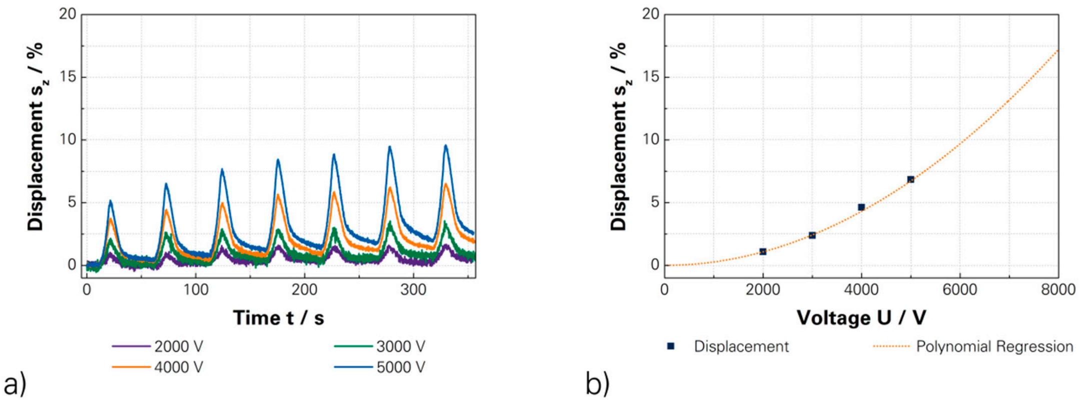

Figure S14. (**a**) Correlation of driving voltage and actuation displacement of a softer DEA (13:1) with an elastic modulus of 0.86 MPa; and (**b**) the calculated characteristic curve—Table S1: actuator 3 [1].

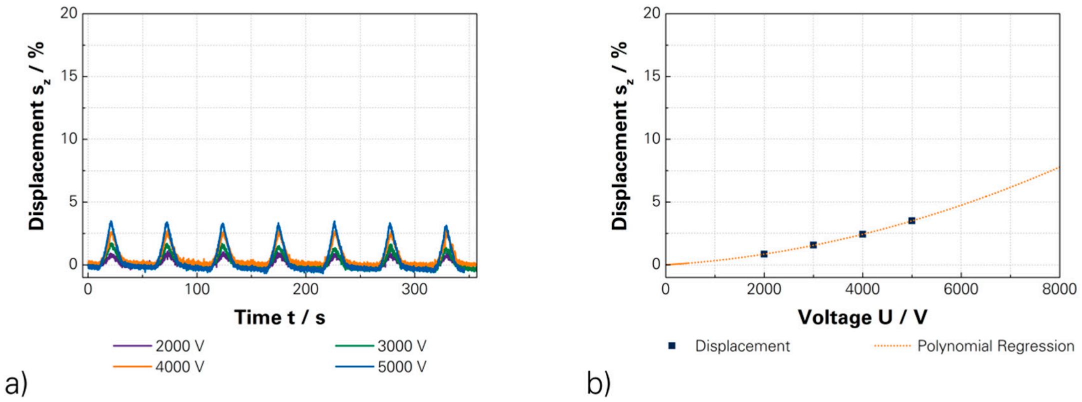

Figure S15. (**a**) Correlation of driving voltage and actuation displacement of a softer DEA (16:1) with an elastic modulus of 0.81 MPa; and (**b**) the calculated characteristic curve—Table S1: actuator 4 [1].

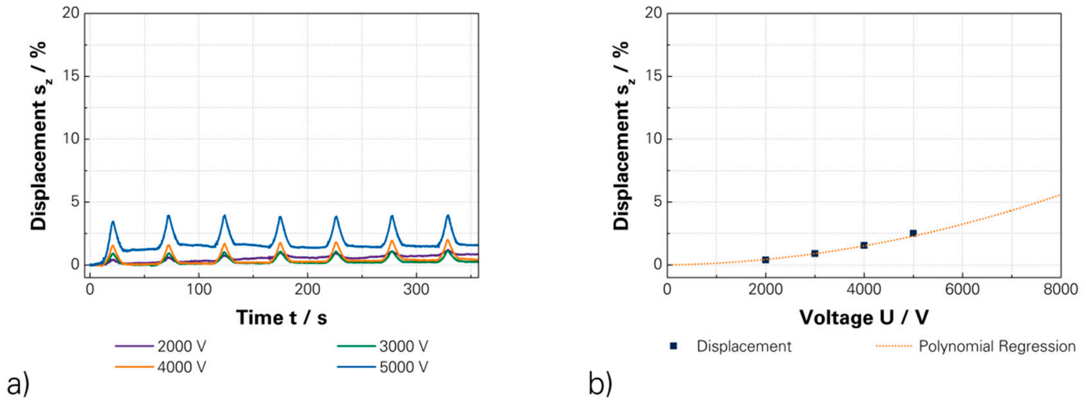

Figure S16. (**a**) Correlation of driving voltage and actuation displacement of a softer DEA (21:1) with an elastic modulus of 0.52 MPa; and (**b**) the calculated characteristic curve—Table S1: actuator 5 [1].

11. Maximum Displacement Regarding Elastic Modulus

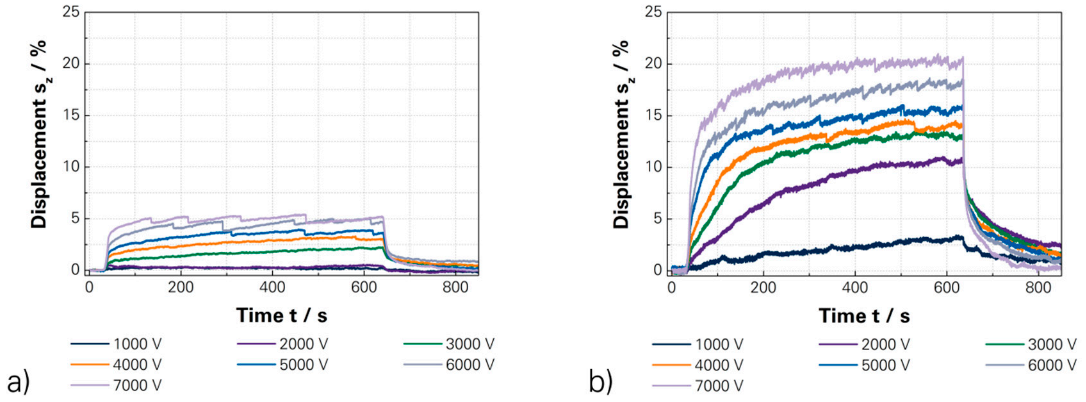

Figure S17. (**a**) Comparison of the maximum displacements of the DEAs with test periods with more than 600 s for three-layer DEAs with raw silicone dielectric and a curing agent ratio of 10:1; and (**b**) 13:1—Table S1 actuators 2 and 3 [1].

12. Actuation Behaviour Regarding Thickness

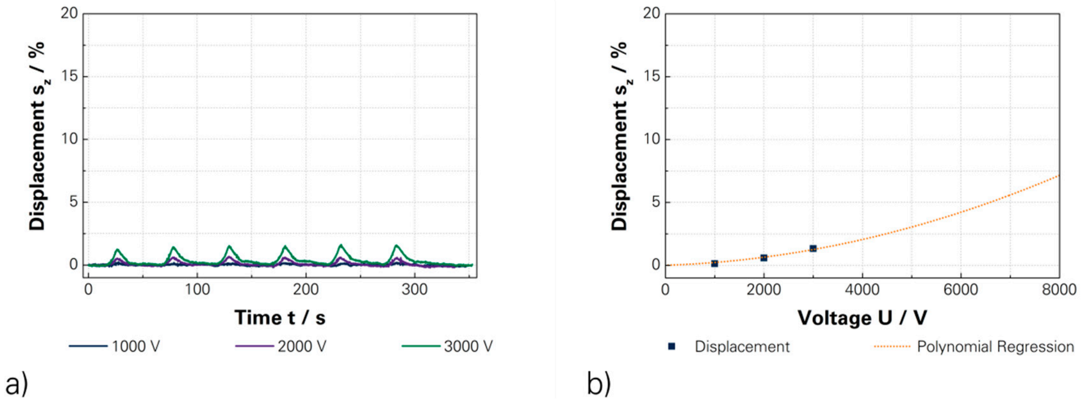

Figure S18. (**a**) Correlation of driving voltage and actuation displacement of a standard DEA (10:1) with a thickness of 105 μm; and (**b**) the calculated characteristic curve—Table S1: actuator 2 [1].

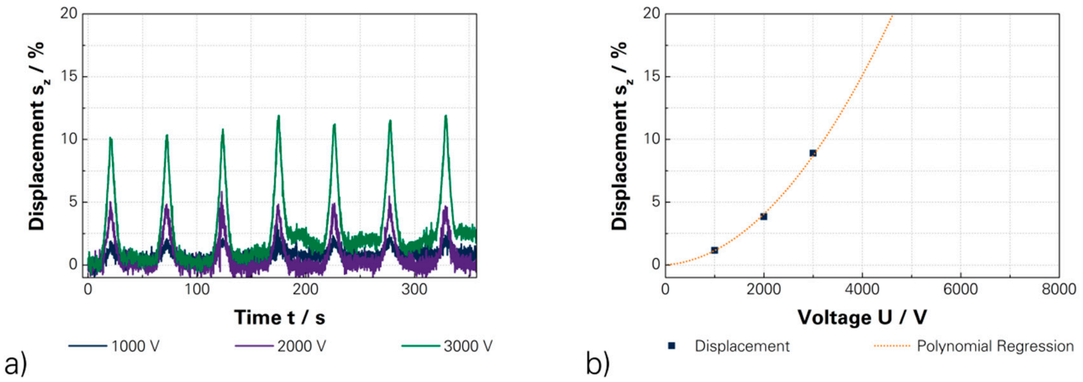

Figure S19. (**a**) Correlation of driving voltage and actuation displacement of a standard DEA (10:1) with a thickness of 46 µm; and (**b**) the calculated characteristic curve—Table S1: actuator 7 [1].

13. Actuation Behaviour Regarding Relative Permittivity

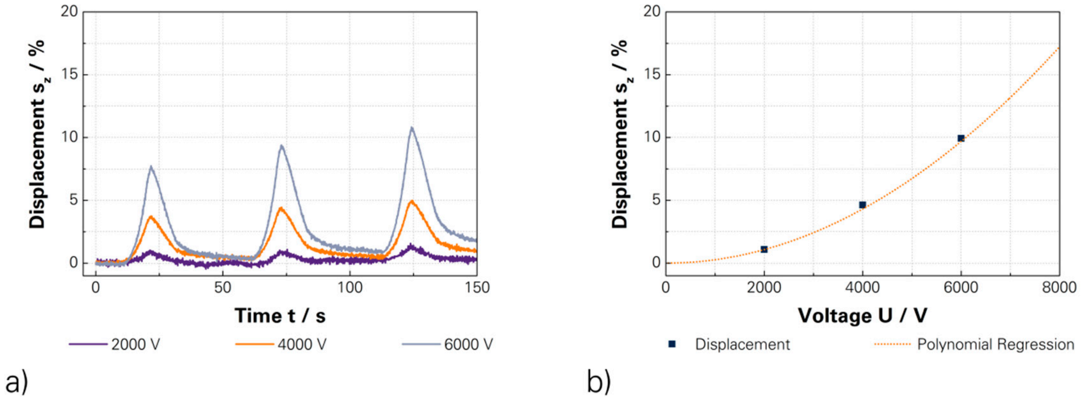

Figure S20. (**a**) Correlation of driving voltage and actuation displacement of a softer DEA (13:1) with a relative Permittivity of 3.24 and an elastic modulus of 0.86 MPa; and (**b**) the calculated characteristic curve—Table S1: actuator 3 [1].

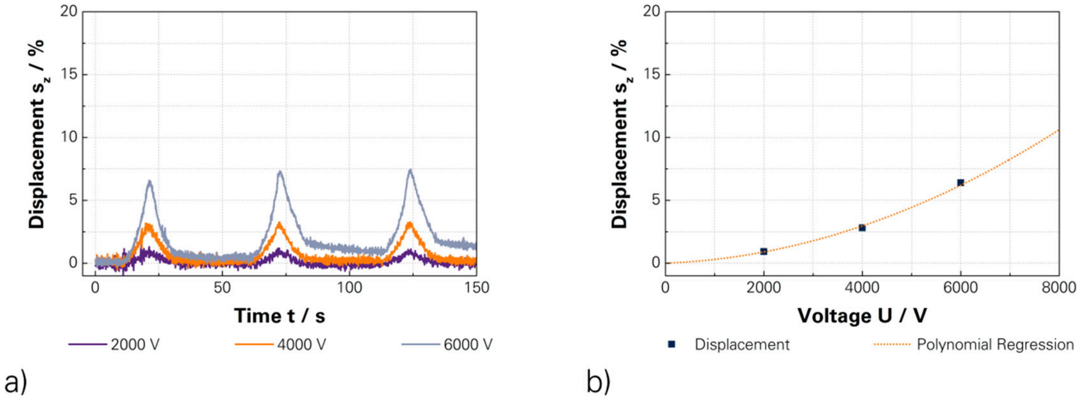

Figure S21. (**a**) Correlation of driving voltage and actuation displacement of a softer DEA (13:1) modified with 33.3 wt % of PMN-PT with a relative permittivity of 4.62 and an elastic modulus of 1.86 MPa; and (**b**) the calculated characteristic curve—Table S1: actuator 8 [1].

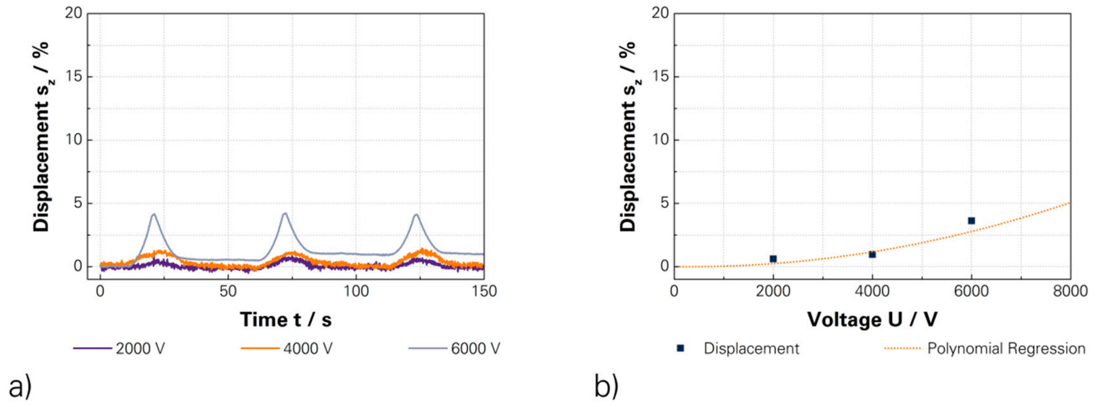

Figure S22. (**a**) Correlation of driving voltage and actuation displacement of a softer DEA (13:1) modified with 50.0 wt % of PMN-PT with a relative permittivity of 6.79 and an elastic modulus of 3.06 MPa; and (**b**) the calculated characteristic curve—Table S1: actuator 9 [1].

14. Actuation Behaviour for Long-Term Exposure

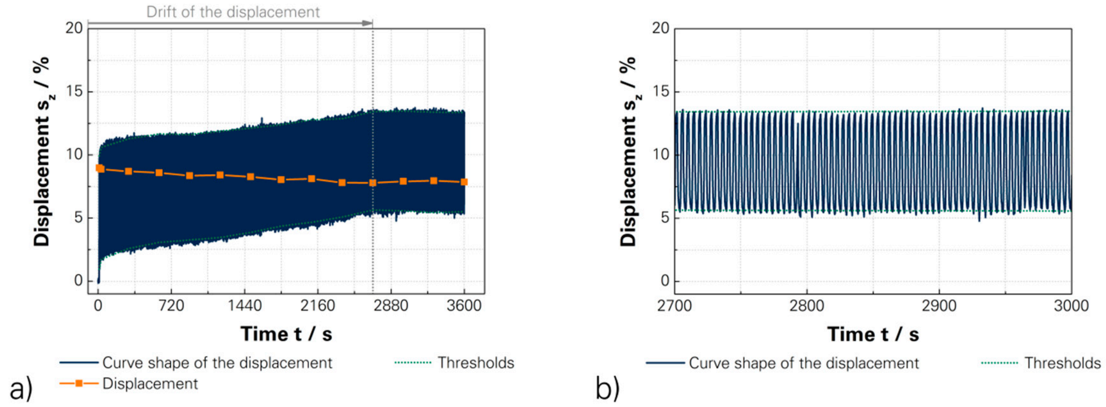

Figure S23. (**a**) Shift of the curve shape of the displacement across time and regarding actuation cycles; and (**b**) a section of continuous working conditions—Table S1: actuator 6.

References

1. Köckritz, T. Entwicklung Neuartiger Elektroaktiver Polymere auf Basis Vollpolymerer Monolithischer Schichtaufbauten. Ph.D. Thesis, TU Dresden, Dresden, Germany, February 2016.
